# Genetic Modification of *mfsT* Gene Stimulating the Putative Penicillin Production in *Monascus ruber* M7 and Exhibiting the Sensitivity towards Precursor Amino Acids of Penicillin Pathway

**DOI:** 10.3390/microorganisms7100390

**Published:** 2019-09-24

**Authors:** Rabia Ramzan, Muhammad Safiullah Virk, Zafarullah Muhammad, Amani Mohedein Mohammed Ahmed, Xi Yuan, Fusheng Chen

**Affiliations:** 1 Hubei International Scientific and Technological Cooperation Base of Traditional Fermented Foods, Huazhong Agricultural University, Wuhan 430070, China; rabiaramzan@webmail.hzau.edu.cn (R.R.); safiullahvirk@yahoo.com (M.S.V.); yuanxiraiden@163.com (X.Y.); 2 College of Food Science and Technology, Huazhong Agricultural University, Wuhan 430070, China; 3 Key Laboratory of Environment Correlative Dietology, Ministry of Education, Huazhong Agricultural University, Wuhan 430070, China

**Keywords:** *Monascus ruber* M7, edible fungi, β-lactam, penicillin G, isopenicllin N, secondary metabolites, major facilitator superfamily transporter, phenylacetic acid, D-valine, L-cysteine

## Abstract

The biosynthesis of penicillin G (PG) is compartmentalized, which forces penicillin and its intermediates to cross the membrane barriers. Although many aspects around the penicillin intermediates traffic system remain unclosed, the transmembrane transporter protein involvement has been only predicted. In the present work, detection of PG and isopenicillin N (IPN) in *Monascus ruber* M7 was performed and functions of *mfst* gene as a transporter were investigated by the combination of gene deletion (Δ*mfst*) complementation (Δ*mfsT*::*mfsT*) and overexpression (M7::PtrpC-*mfsT*). While, the feeding of PG pathway precursor side chain and amino acids, i.e., phenylacetic acid, D-valine, and L-cysteine was performed for the interpretation of *mfsT* gene role as an intermediate transporter. The results showed that, the feeding of phenylacetic acid, D-valine, and L-cysteine possessed a significant effect on morphologies, secondary metabolites (SMs) production of all above-mentioned strains including *M. ruber* M7. The results of UPLC-MS/MS revealed that, Δ*mfsT* interrupt the penicillin G (PG) production in *M. ruber* M7 by blocking the IPN transportation, while PG and IPN produced by the Δ*mfsT*::*mfsT* have been recovered the similar levels to those of *M. ruber* M7. Conclusively, these findings suggest that the *M. ruber* M7 is, not only a PG producer, but also, indicate that the *mfsT* gene is supposed to play a key role in IPN intermediate compound transportation during the PG production in *M. ruber* M7.

## 1. Introduction

*Monascus* spp. are popular in China and other Asian countries as traditional edible fungi, and their fermented products, namely red mold rice (RMR), red yeast rice, *Hongqu* or *Anka*, have been extensively used as folk medicines and food colorants in China for nearly 2000 years [1,2,3,4,5,6]. Nowadays, the researchers have found that *Monascus* spp. can produce various beneficial secondary metabolites (SMs) such as, *Monascus* pigments, monacolin K, γ-aminobutyric acid, and dimerumic acid, and also a harmful SM, e.g., citrinin, a kind of mycotoxin [1,7,8,9,10,11,12]. Recently, Chen. [13] has detected β-lactams in RMR (red mold rice) produced by *M. ruber* M7, and discovered a putative gene cluster responsible for the β-lactam production. He has predicted that the putative β-lactam gene cluster in *M. ruber* M7 is 48-kb in size, comprising the eight genes. Moreover, Wei et al. identified a γ-lactam from RMR by *M. purpureus* [14].

Actually, many microorganisms including Gram negative bacteria, actinomycetes, and filamentous fungi can produce various kinds of lactams, especially β-lactam antibiotics, which have been the cornerstone of antibiotic treatment since the early 1940s [15]. About β-lactam antibiotics biosynthetic pathway, the three precursor amino acids: L-α-aminoadipic acid, L-cysteine, and D-valine, are initially condensed by non-ribosomal peptide synthetase (NRPS), namely δ-(L-α-aminoadipyl)-L-cysteinyl-D-valine synthetase (ACVS) encoded by *pcbAB* gene to form the intermediate δ-(L-α-aminoadipyl)-L-cysteinyl-D-valine (ACV), which is cyclized into isopenicillin N (IPN) by the catalytic activity of isopenicillin N synthase (IPNS) encoded by *pcbC* gene [16]. IPN is a basic nucleus of the β-lactam antibiotics with hydrophilic nature [17]. These steps occur in the cytoplasm of β-lactam producers [18]. Then, IPN can be converted into penicillin G (PG) in *Penicillium chrysogenum* and *Aspergillus nidulans* in the peroxisomal matrix, into cephalosporin C in *Acremonium chrysogenum* in the cytosol and into cephamycin C in *Streptomyces clavuligerus* and *Amycolatopsis lactamdurans* [19,20] in different subcellular compartments. For example, in *P. chrysogenum* the IPN is altered into PG in peroxisomal microbodies, where an isopenicillin N acyl transferase (IAT) catalyzes an α-aminoadipyl side chain (hydrophilic) into a phenyl acetyl CoA (hydrophobic) [21]. Most of the secondary metabolites, as well as, their key enzymes that may cause toxicity to host cells are localized in the microbodies. Such as IAT and phenylacetyl CoA-ligase (PACL) are the crucial enzymes for the PG production, localized in peroxisomes. When the putative targeting signal was removed, the enzyme was not directed to the microbody but instead localized in the vacuole and surrounding cytosol. Under these conditions, production of penicillin was halted [22,23]. The other explanation, that is named as IAT is not able to perform the catalytic reaction in the cytosol due to pH changing effect. Hence, the IPN should be transformed into the peroxisomes for IAT enzyme action and the final production of the benzyl penicillin, phenoxy methyl penicillin. Otherwise production of penicillins in the culture medium might be limited due to the blockage of IPN transportation [24,25]. So the shipping of intermediates and their precursors across the cellular membranes is a fundamental issue during the biosynthesis of penicillins [26], but many transporters remain unexplored [17,27], which include multidrug and toxic compound extrusion, ATP-binding cassette superfamily, small multidrug resistance, major facilitator superfamily (MFS) and so on according to their capabilities to transport different organic composites [26].

Among them, MFS transporters perform a dominant role in many physiological processes of life [28,29,30,31,32]. One MFS transporter gene, *cmcT* is involved in the biosynthesis of cephamicin in *A. lactamdurans* and *S. clavuligerus* [17]. Three MFS transporters genes, *cefT*, *cefP*, and *cefM* take part in the intermediate translocation during cephalosporin C biosynthesis in *A. chrysogenum* [33,34,35,36]. Similarly, Fernández-Aguado et al. discovered two MFS transporters penV and paaT in *P. chrysogenum* to participate the precursor amino acids and phenylacetate translocation, respectively [27,37].

Although many aspects around the penicillin traffic system are still needed to be explored, the transmembrane transporter proteins are supposed to be involved in it. And the strong prediction about the major facilitator superfamily transporter (MFST) encoding genes presence in the gene clusters for the synthesis of the secondary metabolites has been observed (Refs). In current research, an MFS transporter-like protein gene in a putative gene cluster of PG in *M. ruber* M7, called *mfsT* was found by homologous analysis, and then its functions were investigated through *mfsT*-deletion, complementation, and overexpression in the PG pathway.

## 2. Materials and Methods

### 2.1. Materials

*M. ruber* M7 strain (CCAM 070120, Culture Collection of State Key Laboratory of Agricultural Microbiology, Wuhan, China) which has ability to produce the *Monascus* pigments, citrinin but no monacolin K, was used to generate the Δ*mfsT* strain and the M7::PtrpC-*mfsT* strain [38]. The Δ*mfsT* strain was genetically modified to produce the Δ*mfsT*::*mfsT* strain. For the phenotypic analysis, potato dextrose agar (PDA), glycerol nitrate agar (25%) (G25N), malt extract agar (MA) and Czapek yeast extract agar (CYA) media were applied [39]. For transformants screening, 30 μg/mL of hygromycin B and 15 μg/mL of neomycin were utilized as resistant markers on PDA. All strains were maintained at 28 °C on PDA slants.

### 2.2. DNA Extraction

The cetyltri-methylammonium bromide method was used to extract the genomic DNA of strains from the mycelia that were grown on PDA plates covered by cellophane membrane as the detailed procedure has already been described by Shao et al. [40].

### 2.3. mfsT Gene Cloning and Computational Analysis

The primer pairs used in the current study are shown in Table 1. The genomic DNA of *M. ruber* M7 was utilized to amplify the *mfsT* gene by PCR and the conditions were followed as initial denaturation at 94 °C (5 min) succeeding by 35 amplification cycles with the subsequent conditions at 94 °C (30 s), 58 °C (30 s), 72 °C (1 min), while the last extension step at 72 °C (10 min) was performed using T100 Thermal Cycler (Bio-Rad, Hercules, CA, USA) [41,42]. Amino acid sequences encoded by *mfsT* were predicted by the SoftBerry’s FGENESH program (https://linuxl.softberry.com/berry.phtml), and the *mfsT* functional regions were analyzed with the Pfam 27.0 program (http://pfam.xfam.org/). The homology of the deduced amino acid sequences of *mfsT* was interpreted by the BLASTP program (https://blast.ncbi.nlm.nih.gov/Blast.cgi).

### 2.4. Construction of the mfsT Gene Deletion, Complementation, and Overexpression Strains

**Figure 1:**
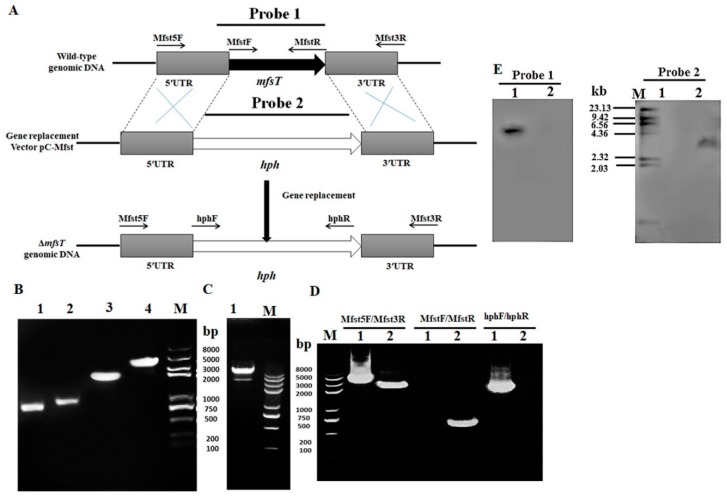
(**A**) Pictorial presentation for the homologous recombination approach to create facilitator superfamily transporter (*mfsT*) disruption strains; (**B**) M: Trans 2k plus II Marker; lane 1: PCR product of 5’ franking region (759 bp); lane 2: PCR product of 3’ franking (677 bp); lane 3: *hph* (2137 bp); lane 4: PCR product of recombinant fragment of 5’UTR, *hph* and 3’UTR (3573 bp); (**C**) lane 1: Restriction enzyme digestion analysis of vector pC-MfsT; (**D**) validation of *mfsT* homologous recombination events; lane 1, the Δ*mfsT* strain; lane 2, the wild-type strain (M7). Different distinct bands obtained by PCR amplification for selected pair of primer; (**E**) southern hybridization analysis; lane 1: Xba1 digested genomic DNA of Δ*mfsT*; lane 2: Xba1 digested genomic DNA of M7, respectively; M: λDNA/*HindIII* marker; probe 1: *mfsT* gene; probe 2: *hph* gene.

The targeted gene *mfsT* deletion, complementation, and overexpression were performed according to the method reported by Shao et al. [40]. The gene disruption cassette (5’UTR-*hph*-3’UTR), complementation cassette and overexpression cassette (5’UTR-PtrpC promoter-*neo*-*mfsT* gene-3’UTR) were constructed by the double-joint PCR method with primer pairs listed in Table 1, and the schematic illustration is shown in Figure 1A [43]. *Agrobacterium tumefaciens* cells containing the disruption vector (pC-MfsT), overexpression vector (pC-OEMfsT) of *MfsT* were constructed and co-cultivated with *M. ruber* M7 to produce the deletion strain (Δ*mfsT*) and the overexpression strain (M7::PtrpC-*mfsT*), respectively. While the *Agrobacterium tumefaciens* cells having vector (pC-OEMfsT) were co-cultivated with the Δ*mfsT* strain to generate the complementation strain Δ*mfsT*::*mfsT*

### 2.5. Southern Hybridization Analysis

The PCR and Southern blot were applied to further confirm the *mfsT* gene deletion, complementation, and overexpression strains using the procedure of the DIG-High Prime DNA Labeling & Detection Starter kit I (Roche, Mannheim, Germany). The DNA of putative Δ*mfsT*, Δ*mfsT*::*mfsT*, and M7::PtrpC-*mfsT* strains, as well as *M. ruber* M7 was digested with restriction enzyme XbaI. By PCR, the amplicons of probe 1 (*mfsT* gene), probe 2 (*hph* gene), and probe 3 (*neo* gene) were amplified with the primer pairs MfsTF/MfsTR, hphF/hphR, and G418F/G418R, respectively (Table 1). The Δ*mfsT* strain was confirmed by probe 1 and 2, Δ*mfsT*::*mfsT* strain by probe 2 and 3, and M7::PtrpC-*mfsT* strain by probe 1 and 3.

### 2.6. Quantitative Real-Time PCR (qRT-PCR) Analysis

qRT-PCR was implemented with the SLAN Fluorescence Quantitative Detection System from Wuhan Good BioTechnology Co., Ltd. (Wuhan, China) by the method as previously described by Liu et al. [44].

### 2.7. Phenotypic Characterization

The parental strain (*M. ruber* M7), Δ*mfsT*, Δ*mfsT*::*mfsT*, and M7::PtrpC-*mfsT* were inoculated on G25N, MA, PDA, and CYA media plates for 15 days at 28 °C to observe the colonial and microbiological features [45].

### 2.8. Biomass Estimation

The biomasses of *M. ruber* M7, Δ*mfsT*, Δ*mfsT*::*mfsT*, and M7::PtrpC-*mfsT* were estimated by using the gravimetric method. The mycelia were collected on PDA plates, then dried at 60 °C until constant weight and the mean biomasses were calculated by three replicates [46].

### 2.9. Extraction and Measurement of the PG Contents

#### 2.9.1. PG Extraction

One mL of the freshly harvested spores (10^5^ cfu/mL) from all transformants and wild-type strain *M. ruber* M7 was respectively grown on PDA plates covered with cellophane membranes and put at 28 °C for 7 days. The PG contents from the mycelia were extracted according to the method described by Senyuva et al. [47] with minor modification. The lyophilized mycelia (0.1 g) were put in acetonitrile (1.5 mL) and 1% formic acid in ethyl acetate (1 mL) and subjected to ultra-sonication for 10 min (KQ-250B, Kunshan, China). After the solvent was evaporated, the left sediment was suspended in 1% (*v*/*v*) formic acid solution, and then filtered through a 0.2-μm membrane before analysis.

From the aqueous phase the PG detection was performed by the method as described by Bi et al. [48] with slight modifications. Aliquots of 2 mL of broth blended with solution of ammonium sulfate of 50% *w*/*v* and polyethylene glycol (PEG) of 50% *w*/*v*, which was added gradually with ratio 1:1. The mixture homogenized by using the vortex used for 20 s and then centrifuged for 10 min at speed 3000× *g*, with temperature 4 °C. Two layers were formed, the upper PEG phase comprising PG, which was separated and analyzed by HPLC.

#### 2.9.2. PG Detected by HPLC

The extracellular and intracellular PG contents were analyzed by HPLC as the method described by Ullán et al. [35]. 20 μL filtrates of the samples were injected in a HPLC (Waters, Milford, MA, USA) with a reverse-phase C18 column (Phenomenex Luna, 250 mm× 5 μm, Phenomenex, Torrance, CA, USA). The gradient elution was performed at flow rate (0.3 mL/min) using methanol as a mobile phase and 5% methanol in 50 mM ammonium formate (pH 3.5) as B mobile phase. A program for the elution gradient was set as follows: From 0 to 8 min the phase A 15% and phase B 85% were used; from 8 to 16 min the phase A 20% and phase B 80% were used, succeeding a washing step with phase A (100%) from 16 to 23 min and a final equilibration step starting at 23 min with 15% B. The temperature for the analytical column was maintained at 35 °C during the whole process. Ultraviolet detection of PG was carried using the 2487 UV/Vis detector at a 240 nm wavelength.

#### 2.9.3. PG and IPN Verified by UPLC-MS/MS

For the mass interpretation by the UPLC, the separation achieved on an Acquity Ultra Performance Liquid Chromatography (UPLC) system (Waters, Milford, MA, USA) with an Acquity BEH C-18 column (2.1 mm × 100 mm, 1.7 μm) with PDA detector, a gradient program for elution employed with the mobile phase combining 25% solvent A (0.1 mM sodium acetate in water) and 55% solvent B (acetonitrile) 20% solvent C was water. The flow rate was set at 0.3 mL/min and the injection volume 2 μL used. The temperature for the column and samples was maintained at 40 °C and 4 °C, respectively.

The mass profile of the extract generated by using the Acquity TQD tandem quadruple mass spectrometer (Waters, Manchester, UK), the appliance functioned with an electrospray ionization (ESI) source. For the MS detection, the conditions for the ESI-MS were used as described by Liu et al. [49]. The PG standard having more than 98.0% purity (Sigma- Aldrich, Saint Louis, MO, USA) was used to approve the PG metabolites existence in the extract [50].

### 2.10. Feeding of Precursor Amino Acids

To evaluate the transportation mechanism of *mfsT* gene the feeding of pathway amino acid was performed [51]. For this step, *M. ruber* M7, Δ*mfsT*, Δ*mfsT*::*mfsT*, and M7::PtrpC-*mfsT* strain cultivated on PDA against the supplementation of pathway side chain and amino acid such as phenylacetic acid, D-valine, and L-cysteine at 2 mM concentration, separately. Further analyzed for biomass production (Section 2.8.), phenotypic characteristics (Section 2.7.), and PG production (Section 2.9.1).

### 2.11. Statistical Analyses

All experiments were analyzed in triplicates. Statistics 8.1 program (Analytical Software, SAS/STAT®, Cary, NC, USA) was utilized for the statistical analyses. In the Tukey test, the *p*-value < 0.05 was considered as statistically significant and *p* < 0.01 as highly significant.

## 3. Results

### 3.1. mfsT Gene Sequence Analysis in M. ruber M7

The putative gene *mfsT* with a 1729 bp fragment size was successfully amplified by utilizing the genomic DNA of *M. ruber* M7. The *mfsT* gene protein sequence from M7 was a blast on NCBI (https://blast.ncbi.nlm.nih.gov/Blast.cgi), and homologous alignment of the MfsT with 25 different MFS transporters from other genomes has been found [52]. As well as, the 527 amino acids sequence was predicted by using the SoftBerry’s FGENESH. A database exploration with Pfam 27.0 program (http://pfam.xfam.org/) exhibited that *mfsT* belongs to the MFS transporter family [53]. As well as, the sequence of MfsT protein was analyzed with the algorithms TMHMM (http://www.cbs.dtu.dk/services/TMHMM/) to determine the number of TMSs (transmembrane spanners) [34]. The results reveal that the MfsT protein possesses 12 hydrophobic TMSs and which arranged into two individually folded domains having six successive TMS, (Supplementary Figure S1.). Similarly, tridimensional conformation modeling of the MfsT protein was created by the SwissProt (http://swissmodel.expasy.org/) [54]. Which displays that 12 α-helixes transmembrane segments surrounding the central substrate binding site in MfsT protein (Supplementary Figure S2.).

### 3.2. Genetic Engineering of mfsT Gene

To investigate the function of *mfsT* in vivo, the development of the Δ*mfsT* gene disruption, complementation, and overexpression strains was done by using a cited method [40,44,55]. Four putative deleted (Δ*mfsT*) strains obtained, which were further identified and validated by PCR analyses. Data related to one mutant has been displayed in Figure 1D, the genomic DNA of Δ*mfsT* strain used as a template for the primer pairs MfsTF/MfsTR (Table 1.) no band of DNA amplified in Δ*mfsT* strain, as compared to *M. ruber* M7, which showed the clear DNA band of size 700 bp. However, *hph* gene with 2137 bp fragment size amplified from Δ*mfsT* and for the *M. ruber* M7 lane remained blanked with the primers hphF/hphR (Table 1.). Additionally, the southern blot analysis was performed to confirm the right homologous recombination in the putative deletion mutants for *mfsT*. A probe 1, which gave a single hybridizing band (5.5 kb) in *M. ruber* DNA digested with the XbaI enzyme. So, the wild type strain possessing a single copy of the gene, Δ*mfsT* lane remained unspotted. Similarly, with probe 2, a band of size 3.8 kb appeared in Δ*mfsT*, which proved that the presence of only one integrated copy of the *mfsT* disruption was constructed and the *M. ruber* M7 remained blank (Figure 1E).

**Figure 2:**
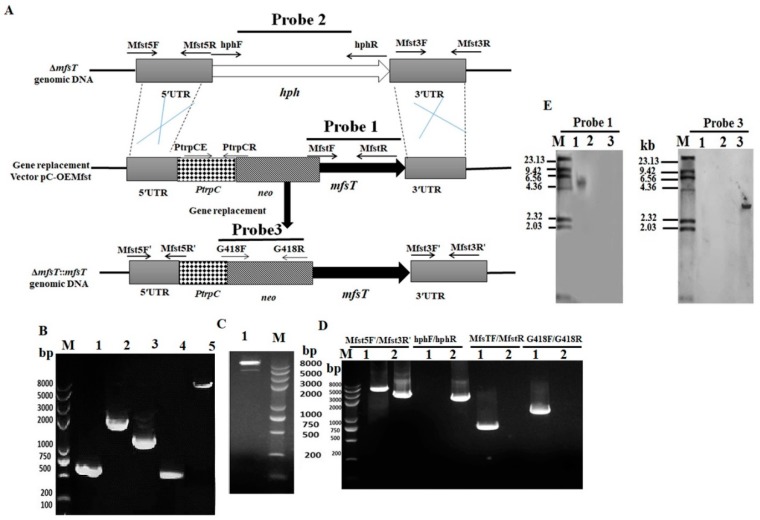
(**A**) Pictorial presentation for the homologous recombination approach to create *mfsT* complement strains; (**B**) M: Trans 2k plus II Marker; lane 1: PCR product of 5’ franking region (761 bp); lane 2: PCR product of 3’ franking (2312 p); lane 3: *neo* (1221 bp); lane 4: PCR product of *PtrpC* promoter (373 bp); lane 5: *mfsT* complementation cassette (4667 bp); (**C**) lane 1: Restriction enzyme digestion analysis of vector pC-OEMfsT for complementation; (**D**) validation of Δ*mfsT*::*mfsT* homologous recombination events. Lane 1, Δ*mfsT*::*mfsT*; lane 2, Δ*mfsT*. Different distinct bands obtained by PCR amplification for selected pair of primer; (**E**) southern hybridization analysis; lane 1: Xba1 digested genomic DNA of Δ*mfsT*; lane 2: Xba1 digested genomic DNA of M7; lane 3: Xba1 digested genomic DNA of Δ*mfsT*::*mfsT*, respectively, M: λDNA/*HindIII* marker; probe 2: *hph* gene; probe 3: G418 gene.

Three presumed neo resistance complementary (Δ*mfsT*::*mfsT*) strains were collected and further investigated. The data associated with one strain has been demonstrated here. During the PCR analyses verification, for the primer pair MfsTF/MfsTR the 0.7 kb size DNA band appeared for Δ*mfsT*::*mfsT* strain, nothing was amplified in Δ*mfsT*. On the opposite, for the primer pairs of G418F/G418R the 1.2 kb fragment amplified in Δ*mfsT*::*mfsT* and the lane for Δ*mfsT* remained blank (Figure 2D). Moreover, to confirm the successful homologous recombination, no DNA band amplified for hphF/hphR primers in Δ*mfsT*::*mfsT* strain, while a band with 2.2 kb appeared in Δ*mfsT*. Hence, the hygromycine B resistance has been replaced by the neomycine resistance successfully (Figure 2D). Further, the genomic DNA of Δ*mfsT*::*mfsT* and Δ*mfsT* amplicons of different sizes for the primers MfsT5F′/MfsT3R′ are shown in (Table 1.) such as, 4.6 kb and 3.5 kb, respectively. The southern blotting revealed that no hybridization band was noticed in Δ*mfsT*::*mfsT*, while a band of size 5.5 kb was observed in Δ*mfsT* for probe 2. Furthermore, to prove the successful homologous recombination, probe 3 gave a 3.5 kb band in Δ*mfsT*::*mfsT* and nothing in Δ*mfsT* (Figure 2E).

**Figure 3:**
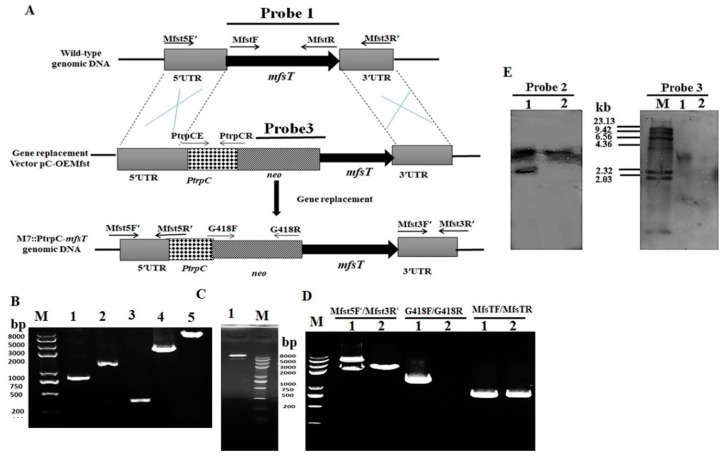
(**A**). Pictorial presentation for the homologous recombination approach to create *mfsT* overexpression strains; (**B**). M: Trans 2k plus II Marker; lane 1: PCR product of 5′ franking region (761 bp); lane 2: PCR product of 3′ franking (2312 p); lane 3: *neo* (1221 bp); lane 4: PCR product of *PtrpC* promoter (373 bp); lane 5: M7::PtrpC-*mfsT* cassette (4667 bp); (**C**). lane 1: Restriction enzyme digestion analysis of vector pC-OEMfsT; (**D**). validation of M7::PtrpC-*mfsT* homologous recombination events. Lane 1, M7::PtrpC-*mfsT*; lane 2, the wild-type strain (M7). Different distinct bands obtained by PCR amplification for selected pair of primer; (**E**). southern hybridization analysis; lane 1: Xba1 digested genomic DNA of M7::PtrpC-*mfsT*; lane 2: Xba1 digested genomic DNA of M7, respectively, M: λDNA/*HindIII* marker; probe 1: *mfsT* gene; probe 3: G418 gene.

Twenty putative M7::PtrpC-*mfsT* strains with neomycin resistance were selected and examined by the PCR system. As shown in Figure 3D, from the genomic DNA of M7::PtrpC-*mfsT* the 1.2 kb product amplified and nothing from a wild strain for the primer pair G418F/G418R. On the other hand, in the case of MfsT5F′/MfsT3R′ primers the two amplicons in M7::PtrpC-*mfsT* appeared with different sizes 4.6 kb and 3.0 kb. In the case of *M. ruber* M7 only a single 3.0 kb band obtained. Which demonstrated that, the M7::PtrpC-*mfsT* carried more than one integrated copy of the PtrpC-*mfsT* overexpression construct (Figure 3D). The Southern blot analysis displayed that, two hybridization bands were noticed in M7::PtrpC-*mfsT* band of size 5.5 kb and 3 kb appeared by probe 1, which confirmed that the overexpression construct (M7::PtrpC-*mfsT*) strain carried only two integrated copies of the *mfsT*. For further confirmation, probe 3 gave a 3.8 kb size band in M7::PtrpC-*mfsT* and nothing in *M. ruber* M7 (Figure 3E).

### 3.3. Real-Time PCR Analysis of ΔmfsT, ΔmfsT::mfsT and M7::PtrpC-mfsT

**Figure 4:**
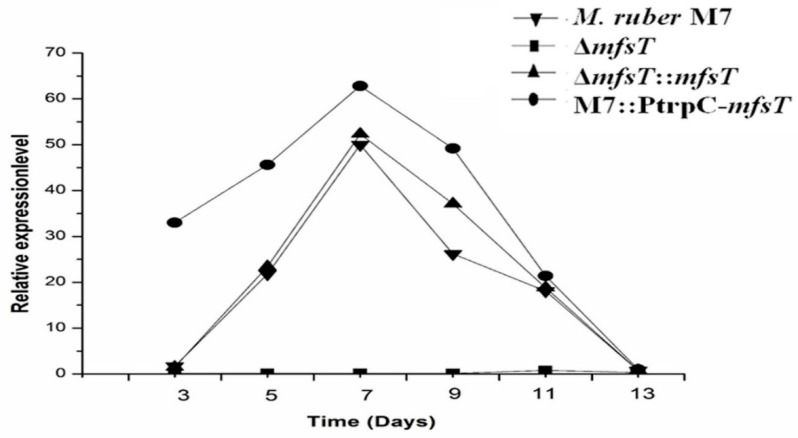
RT-PCR analysis of the *mfsT* gene in the *M. ruber* M7, Δ*mfsT*, Δ*mfsT*::*mfsT*, and M7::PtrpC-*mfsT*. The relative expression 3rd–13th day in *M. ruber* M7, Δ*mfsT*, Δ*mfsT*::*mfsT*, and M7::PtrpC-*mfsT* strains for the *mfsT* gene.

The transcriptional interpretations of the *mfsT* gene was performed by qRT-PCR for all mutant strains such as Δ*mfsT*, Δ*mfsT*::*mfsT*, and M7::PtrpC-*mfsT* as compared to the wild type *M. ruber* M7. As demonstrated in Figure 4, the gene knockout validated in the Δ*mfsT* strain by observing the lowest *mfsT* gene expression. Although, in the *M. ruber* M7 the *mfsT* gene expression level steadily increased and topped at the 7th day, after that the expression level was dropped. While, the *mfsT* expression level was similar in both the complementary strain (Δ*mfsT*::*mfsT*) as well as in the parental strain. In the case of overexpression (M7::PtrpC-*mfsT*), the expression level of *mfsT* was higher compared to that of *M. ruber* M7.

### 3.4. Phenotypic Characterization of ΔmfsT, ΔmfsT::mfsT and M7::PtrpC-mfsT

**Figure 5:**
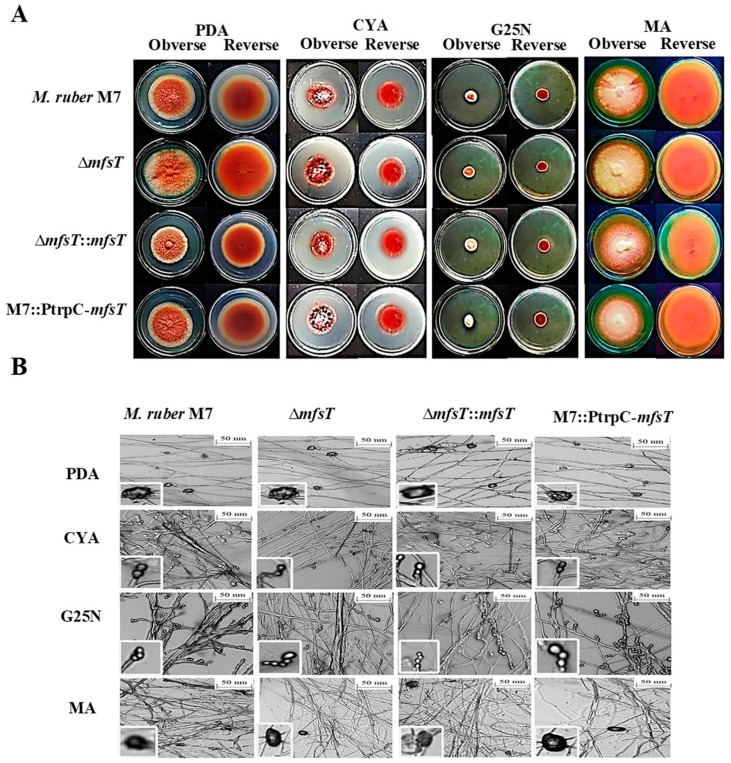
Morphological comparison of *M. ruber* M7, Δ*mfsT*, Δ*mfsT*::*mfsT*, and M7::PtrpC-*mfsT*. (**A**) Colony morphology of *M. ruber* M7, Δ*mfsT*, Δ*mfsT*::*mfsT*, and M7::PtrpC-*mfsT* on PDA, CYA, G25N, MA plates and cultured at 28 °C for 15 days. (**B**) Cleistothecia and conidia development in *M. ruber* M7, Δ*mfsT*, Δ*mfsT*::*mfsT*, and M7::PtrpC-*mfsT* inoculated on PDA, CYA, G25N, MA plates and cultured at 28 °C for 8 days.

To investigate the morphological development variations between the deletion (Δ*mfsT*) strain, complementation Δ*mfsT*::*mfsT* strain, and overexpression M7::PtrpC-*mfsT* strain, as compared to *M. ruber* M7 the phenotypic features were noted. All the mentioned strains inoculated on four distinctive media PDA, G25N, CYA, and MA and incubated for 15 days at 28 °C. While considering the results of *M. ruber* M7 and Δ*mfsT*, Δ*mfsT*::*mfsT*, and M7::PtrpC-*mfsT* the significant difference was observed about the phenotypic analysis, such as, the colony edges, colony appearance size, colony diameter and growth rate among the strains in PDA and MA media plates (Figure 5A). While for G25N the colony color for Δ*mfsT*::*mfsT*, and M7::PtrpC-*mfsT* little bit lighter as compared to *M. ruber* M7 as well as Δ*mfsT*. Moreover, overall development and phenotype of cleistothecia and conidia also demonstrated no variations among the mutant strains as compared to the wild strain (Figure 5B).

### 3.5. Biomass

**Figure 6:**
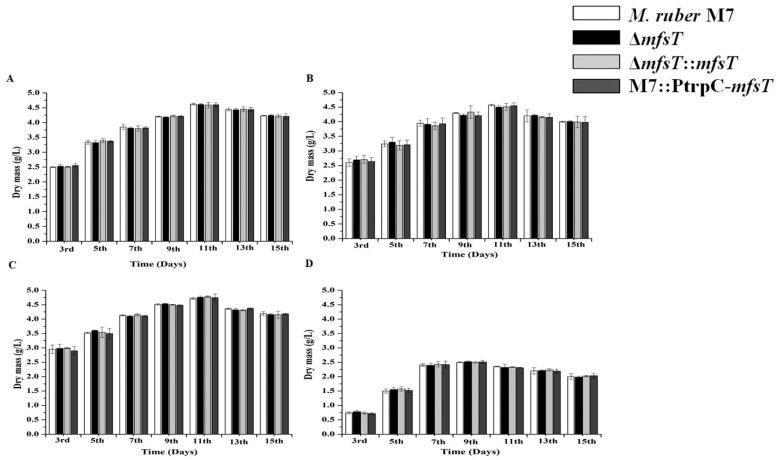
Comparison of biomass (dry cell weight) of the *M. ruber* M7, Δ*mfsT*, Δ*mfsT*::*mfsT*, and M7::PtrpC-*mfsT* against pathway side chain and amino acid. (**A**) PDB medium without amino acid (control); (**B**) PDB medium with D-valine; (**C**) PDB medium with phenylacetic acid; (**D**) PDB medium with L-cysteine, at 2 mM concentrations and incubated at 28 °C without agitation. The bar representing the mean of triplicate values and error bars show standard deviation.

The biomass of *M. ruber* M7 as a control to Δ*mfsT*, Δ*mfsT*::*mfsT*, and M7::PtrpC-*mfsT* strains measured by weighing the dry cell weight of the mycelia. The results presented in Figure 6, showed the significant (*p* < 0.05) increase in the biomass value for all the strains up to the 15th day. However, the greatest biomass was observed on the 11th day and after that overall a decreasing trend in biomass was noted.

### 3.6. Analyses of MP Production ΔmfsT, ΔmfsT::mfsT and M7::PtrpC-mfsT

**Figure 7:**
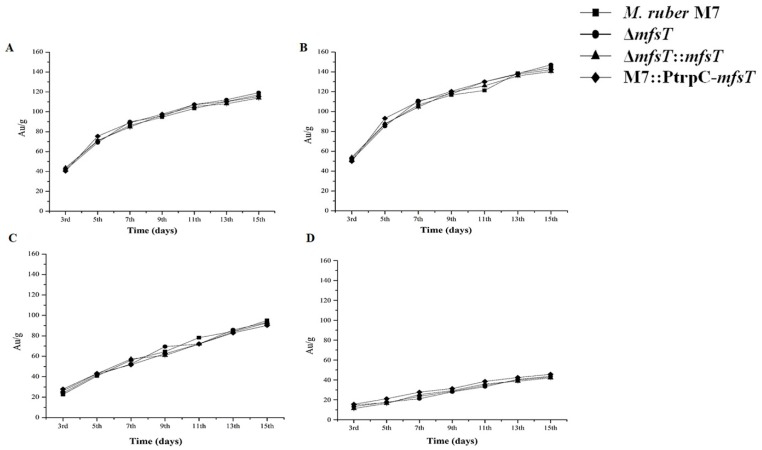
Comparative analysis for the pigments in *M. ruber* M7, Δ*mfsT*, Δ*mfsT*::*mfsT*, and M7::PtrpC-*mfsT* against pathway side chain and amino acid. (**A**) PDB medium without amino acid (control); (**B**) PDB medium with D-valine; (**C**) PDB medium with phenylacetic acid; (**D**) PDB medium with L-cysteine, at 2 mM concentrations and incubated at 28 °C without agitation. (Mean ± sd).

Previously, Liu et al. [44] and Feng et al. [7] reported that *Monascus* spp. can produce many secondary metabolites especially MPs such as red, orange, and yellow. Overall production of the pigments notably (*p* < 0.01) increased from 3^rd^ to 15^th^ day for all strains (Figure 7).

### 3.7. Detection and Production of PG by HPLC and UPLC

The role of the *mfsT* gene in the putative penicillin biosynthesis in *M. ruber* M7, all transformants strains deletion (Δ*mfsT*), complementation (Δ*mfsT*::*mfsT*), and overexpression (M7::PtrpC-*mfsT*) were investigated by the HPLC. All the strains fermented in penicillin production conditions for 7 days. The samples collected at the 7th day beside the treated samples and studied by HPLC for the estimation of the PG.

**Figure 8:**
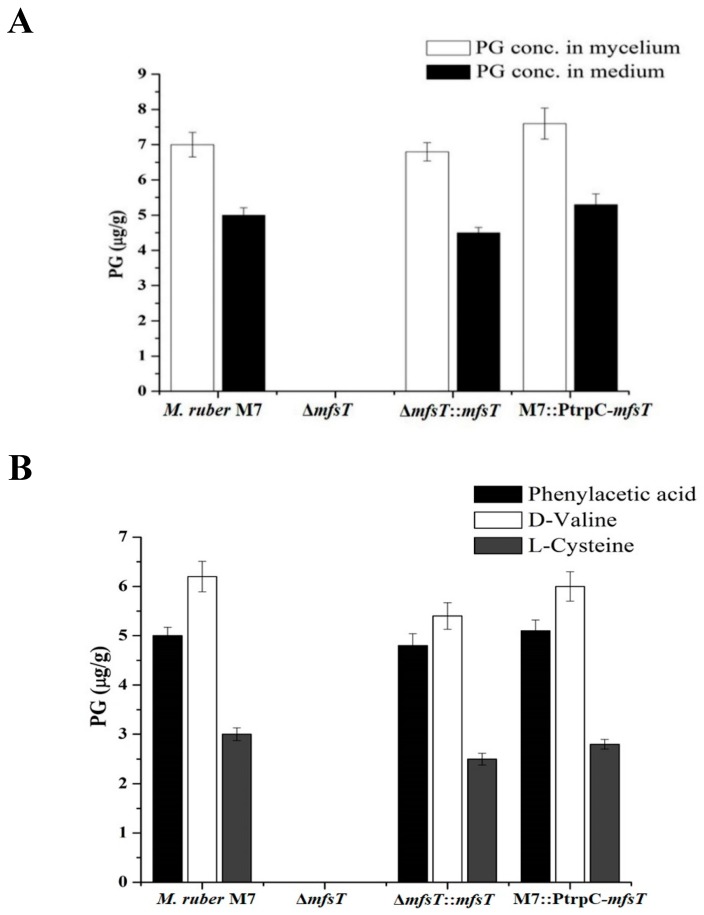
Comparison of PG concentration of the wild-type strain and the *mfsT* transformants at the 7th day. (**A**) PG concentration in mycelium and medium; (**B**) comparison of PG concentration affected by supplementation of D-valine, phenylacetic acid, L-cysteine at 2 mM. The bar representing the mean of triplicate values and error bars show standard deviation.

In the Δ*mfsT* strains, the insufficiency of the extracellular PG was observed, may be due to its accumulation within the cells of the mutant. However, HPLC results indicated that these mutants could not accumulate the intracellular PG (Figure 8A). In these transformants, intracellular PG levels were lower than the detectable levels during fermentation.

The detected PG production summaries among the mutant strains, some of them exhibited an increase such as overexpression (M7::PtrpC-*mfsT*), while Δ*mfsT* revealed no PG production in contrast to the parental strain M7 at the 7th day (Figure 8A). The mean PG yield in intracellular portion of transformants Δ*mfsT*, Δ*mfsT*::*mfsT*, and M7::PtrpC-*mfsT* calculated as 0.0 ± 0.00 μg/g, 6.8 ± 0.26 μg/g, and 7.6 ± 0.43 μg/g, compared to the parental strain, i.e., 7.0 ± 0.35 μg/g (Figure 8A). Whereas, the extracellular results gave the same pattern for the production of PG Δ*mfsT*, Δ*mfsT*::*mfsT*, and M7::PtrpC-*mfsT* yielding the PG, 0.0 ± 0.00 μg/g, 4.5 ± 0.15 μg/g, and 5.3 ± 0.30 μg/g, compared to the parental strain, i.e., 5.0 ± 0.21 μg/g (Figure 8A).

Conclusively, there is clear proof about the Δ*mfsT* gene role for having the depilatory effect on the PG production by blocking transportation of intermediates. While, for further confirmation of the complementation strain, Δ*mfsT*::*mfsT* showed almost similar production rates as did the parental strain.

**Figure 9:**
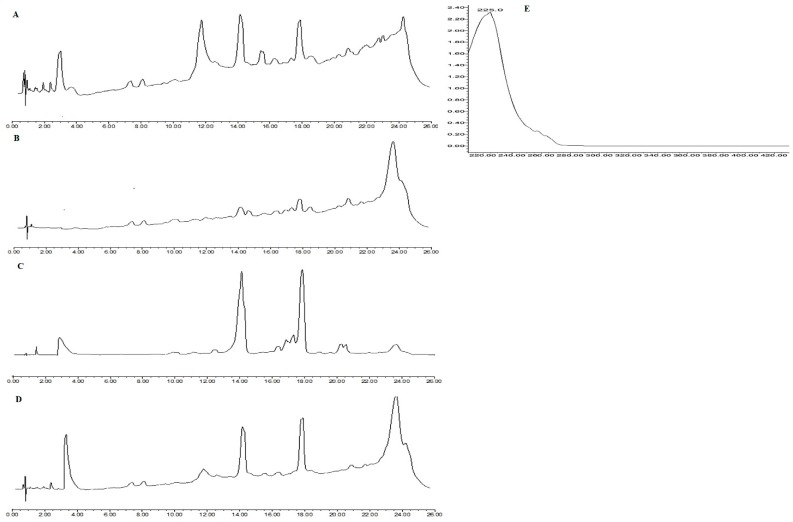
Comparison of eluted products of *M. ruber* M7, Δ*mfsT*, Δ*mfsT*::*mfsT*, and M7::PtrpC-*mfsT* for solid state fermentation on rice. (**A**) Metabolic profile of *M. ruber* M7; (**B**) metabolic profile of Δ*mfsT*; (**C**) metabolic profile of Δ*mfsT*::*mfsT*; (**D**) metabolic profile of M7::PtrpC-*mfsT* at 250 nm. (**E**) UV-Vis spectrum of benzylpenicillin (PG).

The PG presence in the filtrate of *M. ruber* M7, Δ*mfsT*, Δ*mfsT*::*mfsT*, and M7::PtrpC-*mfsT* confirmed by UPLC analyses (Figure 9). The spectrum of penicillin G is presented in (Figure 9E). The peak for penicillin G appeared at approximately 3.8 min in the case of sample filtrate for *M. ruber* M7(Figure 9A), Δ*mfsT*::*mfsT* (Figure 9C), M7::PtrpC-*mfsT* (Figure 9D), having the same spectrum as PG. And no peak appeared in the case of Δ*mfsT* (Figure 9B), which confirmed that none was produced by Δ*mfsT*. Hence, *mfsT* is responsible for controlling the production of PG by transportation of intermediate compounds in *M. ruber* M7. For more confirmation feeding of precursor amino acids has been performed to check the *mfsT* gene function for the transportation of intermediate.

### 3.8. Effect of Feeding of Precursor Amino Acids

First of all, the morphological sensitivity for colony development and SMs of the *M. ruber* M7, Δ*mfsT*, Δ*mfsT*::*mfsT*, and M7::PtrpC-*mfsT* strains were investigated against the phenylacetic acid, D-valine, and L-cysteine The effect on the formation of cleistothecia and conidia was also observed. In the case of phenylacetic acid, D-valine, and L-cysteine feeding fermentation, a sharp decline in the biomass was observed for Δ*mfsT*, Δ*mfsT*::*mfsT* and M7::PtrpC-*mfsT* as compared to the M7 up to a 15th day for all amino acids. The amino acids and side chain feeding exhibited a clear biomass reduction L-cysteine < phenylacetic acid < D-valine. The lowest biomass was observed in L-cysteine feeding experiment (Figure 6B–D).

Despite, the amino acid supplementation affected the ability of the production of the pigments. D-valine feeding significantly increased the yield of pigments in all strains (Figure 7B). While for the exposure of the L-cysteine showed a significant reduction (*p* < 0.01) in the pigment contents for all mutant strains as well as in M7 (Figure 7D). In the case of phenaylacaetic acid supplementation, slower growth has been observed among Δ*mfsT*, Δ*mfsT*::*mfsT*, M7::PtrpC-*mfsT*, and *M. ruber* M7.

Similarly, no PG production was observed in the Δ*mfsT* mutant 0.0 ± 0.00 µg/g (Figure 8B). Hence, from the above results it noted that gene *mfsT* did not involve in the pathway amino acid transportation. While, in other mutants all amino acid supplementation reduced the PG production due to lower biomass yield.

**Figure 10:**
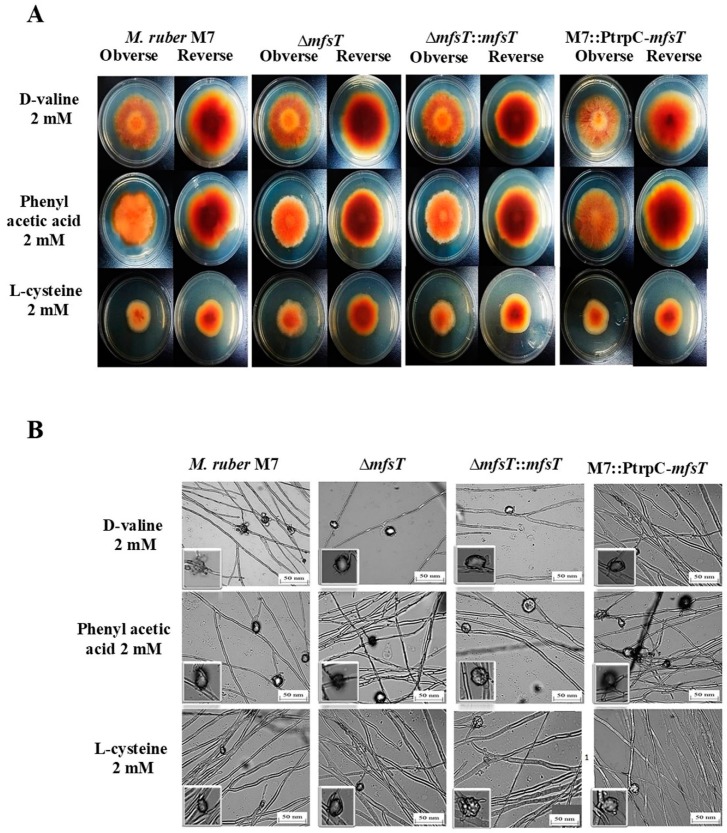
Morphological comparison of *M. ruber* M7, Δ*mfsT*, Δ*mfsT*::*mfsT*, and M7::PtrpC-*mfsT* to evaluate the sensitivity towards pathway amino acid supplementation. (**A**) Colony morphology of *M. ruber* M7, Δ*mfsT*, Δ*mfsT*::*mfsT*, and M7::PtrpC-*mfsT* inoculated on PDA plates which supplemented with D-valine, phenylacetic acid, L-cysteine at 2 mM concentrations for each and cultured at 28 °C for 15 days; (**B**). cleistothecia and conidia development in *M. ruber* M7, Δ*mfsT*, Δ*mfsT*::*mfsT*, and M7::PtrpC-*mfsT* cultured on PDA plates which supplemented with D-valine, phenylacetic acid, L-cysteine at 2 mM concentrations for each and cultured at 28 °C for 8 days.

The effect of pathway amino acids and side chain (phenylacetic acid, D-valine, and L-cysteine) on colony morphology and the formation of the spores of Δ*mfsT*, Δ*mfsT*::*mfsT*, M7::PtrpC-*mfsT* and M7. All strains cultivated on PDA plates which were supplemented with 2 mM concentration of each amino acid and incubated for 15 days at 28 °C. As a result, a significant difference related to diameter and colony morphology was observed among supplemented amino acids. Moreover, the mutants Δ*mfsT*, Δ*mfsT*::*mfsT*, M7::PtrpC-*mfsT* and wild type *M. ruber* M7 colony characteristics change such as, the size, color, and shape (Figure 10A). The phenylacetic acid supplementation decreased the sensitivity among the Δ*mfsT*, Δ*mfsT*::*mfsT*, and M7::PtrpC-*mfsT* strains, the irregularity was observed in colony edges. For L-cysteine, the overall colony diameter and growth rate was decreased in all mutant strains Δ*mfsT*, Δ*mfsT*::*mfsT*, and M7::PtrpC-*mfsT* as compared to the M7. While similar to M7, the change in color of the colony was observed by the feeding of the D-valine in Δ*mfsT*, Δ*mfsT*::*mfsT*, and M7::PtrpC-*mfsT*. However, there was no difference observed in the conidia formation for all strains (Figure 10B). UPLC-MS/MS was performed for further clarification of the function of *mfsT* gene.

### 3.9. Detection of Beta Lactam Metabolites by UPLC-MS Results

**Figure 11:**
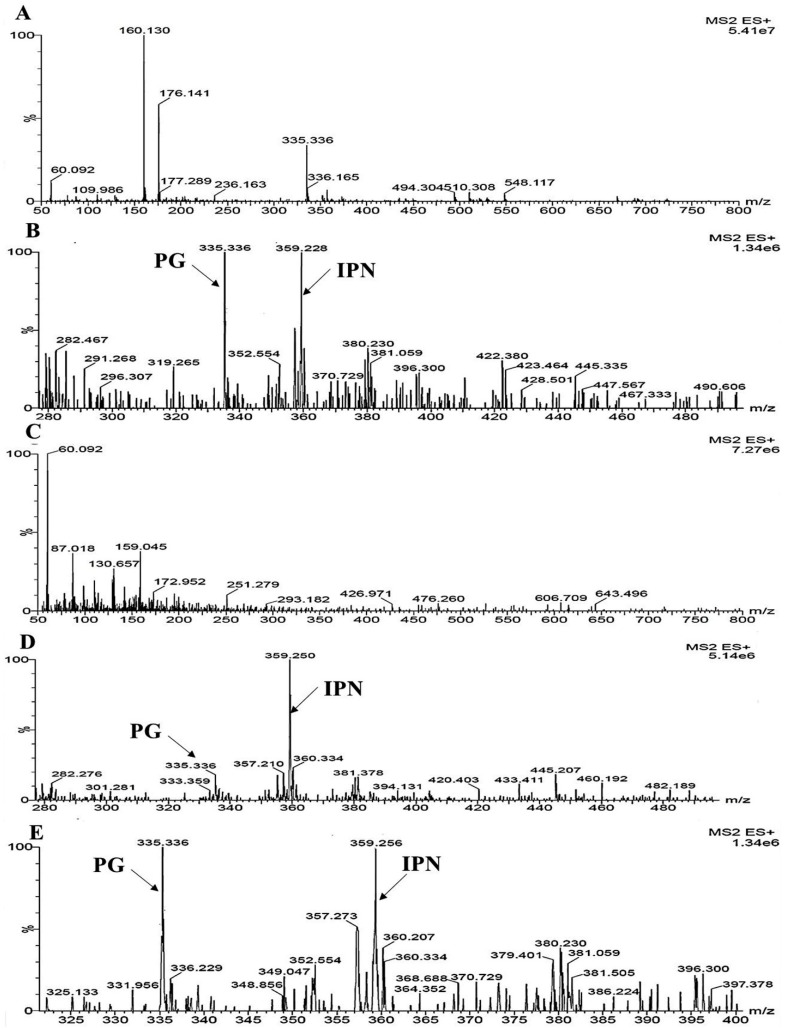
UPLC-MS/MS ([M-H]^−^) spectrogram of the *M. ruber* M7, Δ*mfsT*, Δ*mfsT*::*mfsT*, and M7::PtrpC-*mfsT* for solid state fermented on rice displays that the atomic mass of [M-H]^−^ ion (m/z 335.336) matched well with the standard penicillin. The molecular weight for isopenicillin N is 359.3 g/mol, in spectrogram [M-H]^−^ ion m/z 359.00 representing the isopenicillin N peak. (**A**) Spectra in: A penicillin G (PG) standard with the mass 335.336; (**B**) a pattern of fragmentation of *M. ruber* M7; (**C**) a pattern of fragmentation of Δ*mfsT*; (**D**) a pattern of fragmentation of Δ*mfsT*::*mfsT*; (**E**) a pattern of fragmentation of M7::PtrpC-*mfsT*.

A mass profile of the *M. ruber*, Δ*mfsT*, Δ*mfsT*::*mfsT*, and M7::PtrpC-*mfsT* on solid state fermentation rice filtrate created to explore the metabolites. Since this filtrate indicated a broader range of biological activities. The results of the foot-printing of the abundant metabolites present in *M. ruber* M7 have been presented in Figure 11.

Molecular masses related to the beta-lactam like PG established in the filtrate of the culture. Hence, the PG presence in *M. ruber* M7 extract was also demonstrated in the analysis of UPLC-MS/MS (Figure 11). The occurrence of the mass (335.336 g/mol) in the crude extract of M7 (Figure 11B) and the similar fragmentation pattern exists in the standard penicillin G (Figure 11A) approved the identity of this metabolite. While in the case of Δ*mfsT* no PG peak detected (Figure 11C).

While comparing the results for all strains, another peak for 395 g/mol [56] for isopenicillin N missed in Δ*fsmt* (Figure 11C) as compared to other strains such as *M. ruber*, Δ*mfsT*::*mfsT*, and M7::PtrpC-*mfsT*. Hence, the *mfsT* may be responsible for the transportation of IPN and in deletion mutant (Δ*mfsT*) IPN transportation blocked, which may interfere the PG pathway.

## 4. Discussion

A large number of transporter proteins perform a leading role to maintain the physiological balance from prokaryotic to eukaryotic organisms by permitting the substrate transportation across the lipid bilayers of every cell. From the genomic analysis, 10% of all these transporter proteins belong to the MFS [17,57]. Until now, the secondary metabolites shipping from cellular compartments to export cell are not fully known in fungal cells. Although, the strong prediction reported regarding the presence of a gene encoding the major facilitator superfamily (MFS) transporter proteins in the gene clusters of secondary metabolites. Therefore, in biosynthetic pathways, may be these membrane-spanning proteins take part in compartmentalization or localization between the microbodies or to the extracellular [9,58]. The MFS transporter proteins acted as a secondary carrier operated by the proton (H^+^) motive force created by the electrochemical proton gradient across the transmembrane [25]. It is assumed that as a consequence of the selective evaluation, the MFS transporters undergo the mutation process, which modifies their binding ability to the specific ligand and let them transport the secondary metabolites [59].

In the present exploration, we have analyzed one of the major facilitator superfamily (MFS) member exited in *M. ruber* M7, we named it as “*mfsT*”. The *mfsT* gene responsible for encoding the protein MfsT in *M. ruber* M7 was found similar to *Aspergillus* and *Penicillium* spp. through comparative analyses of their protein sequences with the algorithm Blastp (protein–protein alignment with Basic Local Alignment Search Tool, (NCBI). Supporting this fact, *MrMfsT* (*Monascus ruber* major facilitator superfamily) exhibits the significant amino acid alignment with other MFSs transporter protein related to *Penicillium* species (Table 2.) such as, *chrysogenum* (KZN94365.1), *occitanis* (PCG89412.1), *expansum* (XP_016598554.1), *italicum* (KGO74874.1), *griseofulvum* (KXG49310.1), *digitatum* Pd1 (XP_014538707.1). Typically, most of the MFS transporters possess a 12–14 hydrophobic transmembrane spanners, divided into two specific folded domains with equal number of spanners [59]. The MfsT protein carries two discrete blocks of domain with 6TMS in each domain (Supplementary Figure S1.) Moreover, 6TMS/6TMS domain structure typical characteristic of the MFS members [58]. The hydrophobic binding chamber of MFS transporter undergoes a series of the mutation processes during evolution, which increase its specificity towards the particular secondary metabolites [59]. The binding site of hydrophobic chamber is surrounded by the 12TMS topologically, as predicted by tridimensional modeling (Supplementary Figure S2.).

The participation of *mfsT* in the putative biosynthesis of penicillin G in *M. ruber* confirmed by investigating the three types of mutated strains was achieved by the deletion, overexpression, and complementation of the *mfsT* gene. The experimentation analysis of all types of transformants reveals that the modulations in the *mfsT* gene expression caused the blocked (Figure 9B), increased and, clearly complementary effect on the PG production (Figure 9C).

Ullán et al. found that, in *P. chrysogenum*, the PenM belonged to the MFS transporter protein involved for the passing of substrate IPN across the hydrophobic membranes, because, IPN has a hydrophilic nature that hinders its transportation through the cell [34]. Penicillin N (PenN) is biochemically similar to the IPN and they are chemically enantiomers (L and D) in structure. PenN also acts as a substrate for the CefM another MFS transporter protein of *A. chrysogenum*. This fact points out a conserved range of specificity of these transporter proteins among these two fungi that share part of the beta-lactam biosynthetic pathway [25,60]. Similarly, intermediate compounds of the cephalosporin C transportation encoded by another MFS *cefT* gene [60]. Moreover, it was also predicted that *afTl gene* of aflatoxin biosynthetic gene cluster in *Aspergillus parasiticus* encoded the MFS transporter may affect the production of aflatoxin which is statistically different on the basis of semi quantitative analysis [61,62].

Fernández-Aguado et al. stated that the CmcT which is the MFS transporter protein encoded by the *cmcT* gene putatively involved for intermediate transportation in the biosynthesis cluster of cephamicin in the *A. lactamdurans* and *S. clavuligerus* [17]. A decade ago, by molecular and biochemical working about the biosynthesis of cephalosporin C on *A. chrysogenum* C10 (ATCC 48272), three genes *cefT, cefP, cefM* identified that encoded the MFS transporter proteins in the beta-lactam gene cluster. The gene *cefT* controlled those β-lactams secretion, which have α-aminoadipic derived side chains such as, IPN, PenN, and deacetyl cephalosporin C [33,34,35]. The *cefP* involved in the IPN translocation to the peroxisomal lumen from the cytosol [34]. Similarly, *cefM* involved in the PenN transportation from the lumen to the cytosol of the microbodies. Moreover, the *penV* encoded the membrane MFS transporter genes, which supplies the precursor of the amino acids from the vacuolar pool for the tripeptide ACV formation [37]. The *paaT* gene in *A. chrysogenum* participated in the phenylacetate translocation in the peroxisomal bodies, after internalization of phenylacetate it supplied as a side chain for the PG formulation [27]. Meanwhile, in another report about the *P. chrysogenum* for the transporter ABC40 exhibited the extrusion system to the sorbate, phenylacetate, and benzoate, as well as, shielding the cells from the injurious acidification through the weak acid incorporation to the β-lactam pathway [63].

L-α-aminoadipic acid, L-valine, and L-cysteine have been reported as precursors of penicillin biosynthesis. The cysteine and valine have physiological importance due to their participation in the protein synthesis. While the L-α-aminoadipic acid obtained from the lysine pathway as an intermediate compound. The β-lactam producer strains can produce the higher L-α-aminoadipic acid. Valine acts as the main precursor constituent of the penum nucleus structure [64]. L-valine performed a pivotal function to improve penicillin V (penV) production [65]. There was positive correlation reported in the penV production and L-valine supplementation concentrations in the fermentation medium. Similarly, it was noticed that the enzyme activity of the penicillin biosynthesis was altered by the addition of PAA (phenylacetic acid) into the fermentation medium [66]. The PAA showed an inhibition effect on the biomass yield of *Bacillus badius*. The PAA as a weak acid increases the medium acidity that affects the growth and ultimately lowers the PG production.

## 5. Conclusions

In this study, we cloned and categorized an *mfsT* gene from the *M. ruber* M7 that encoded the MFS transporter. Our outcomes determined that *mfsT* putatively involved in penicillin G (PG) production, possibly by translocation of the intermediate compound IPN across the microbodies. This has been detected for the first time that, the MFS transporter is linked with isopenicillin N transportation in *M. ruber* M7. In the UPLC MS/MS results, the peak appeared with mass 335.336 g/mol and 359.220 g/mol in *M. ruber* M7, Δ*mfsT*::*mfsT*, and M7::PtrpC-*mfsT* except in the Δ*mfsT*. The feeding experiments of the PG precursor amino acids and side chain including phenylacetic acid, D-valine, and L-cysteine showed a significant effect on morphologies, and MPs production of Δ*mfsT*::*mfsT* and M7::PtrpC-*mfsT* including *M. ruber* M7. For the PG results, the pattern for production remains the same for pathway amino acid feeding. Moreover, in *M. ruber* M7 the secretion of PG might have resulted from the supportive activity of the multiple transporters as a transporter.

## Figures and Tables

**Table 1:** Primers used for experimentation.

Name	Sequence 5′⟶3′	Description
**MfsT5F**	GCTCTAGAAGTCCGAGCGCTGCAGC	For the amplification of the 759 bp of the 5′ flanking region of the *mfsT* gene
**MfsT5R**	CAATATCATCTTCTGTCGACTCGGTCTCGCGTGCTTG	
**MfsT3F**	GAGGTAATCCTTCTTTCTAGTTACCCATCAAGGCAGGC	For the amplification of the 677 bp of the 3′ flanking region of the *mfsT* gene
**MfsT3R**	GGGGTACCCCGGAAAGCAGAAGACCA	
**hphF**	GTCGACAGAAGATGATATTG	For the amplification of the 2317 bp of the *hph* cassette from the plasmid pSKH
**hphR**	CTAGAAAGAAGGATTACCTC	
**MfsTF**	GCCCTGTGTGTATTGCTC	For the amplification of the 700 bp of the partial *mfsT* gene which was used as probe 1
**MfsTR**	GTAACCACGGTCCATGAG	
**MfsT5F′**	GCTCTAGATGGCTGGTGCTGAGGGTAG	For the amplification of the 761 bp of the 5′ flanking region for overexpression and complementation of the *mfsT* gene
**MfsT5R′**	CAATATCATCTTCTGTCGACTAGGGCTAAGGGCAAGGGC	
**PtrpCF**	GTCGACAGAAGATGATATTG	For the amplification of the 373 bp of the *trpC* promoter from the plasmid pSKH
**PtrpCR**	GGTTCGGTTCGCATATCGATGCTTGGGTAGAATA	
**G418F**	CCAACTCAACCCCATCGAACCGTAACC	For the amplification of the 1221 bp of the *neo* cassette from the plasmid pKN1
**G418R**	ACGATGCTGGACGGGGACATCATCATGCAACATGCATG	
**MfsT3F′**	GTCCCCGTCCAGCATCGT	For the amplification of the 2312 bp of the 3′ flanking region + ORF for overexpression and complementation of the *mfsT* gene
**MfsT3R′**	GGGGTACCAGCGGCTGGGTAGAGTCC	
**GAPDHF**	CTATGCGTGTGCCTACTTCCA	For the real-time RT-PCR analysis of *gpd*
**GAPDHR**	GAGTTGAGGGCGATACCAGC	
**MfsTF**	TCGTGCTCTCCTTGGGCTTC	For the real-time RT-PCR analysis of *mfsT*
**MfsTR**	TGACGAGAGAGCGGATGAGATT	

**Table 2:** Significative homologous alignment of MfsT protein with other fungi.

Protein Number	Strain	Function	Positive Amino Acid (%)
**XP_001270324.1**	*Aspergillus clavatus NRRL 1*	MFS transporter putative	83
**RDW58813.1**	*Coleophoma cylindrospora*	MFS transporter-10	74
**GAD96209.1**	*Byssochlamys spectabilis No. 5*	MFS transporter putative	57
**XP_016598554.1**	*Penicillium expansum*	Major facilitator superfamily domain, general substrate transporter	54
**KGO74874.1**	*Penicillium italicum*	Major facilitator superfamily domain, general substrate transporter	53
**XP_014538707.1**	*Penicillium digitatum Pd1*	MFS multidrug transporter, putative	55
**KZN94365.1**	*Penicillium chrysogenum*	Proton-coupled folate transporter	53
**KXG49310.1**	*Penicillium griseofulvum*	Major facilitator superfamily domain, general substrate transporter	52
**KFH48645.1**	*Acremonium chrysogenum*	Putative transporter like protein	52
**PCG89412.1**	*Penicillium sp. ‘occitanis’*	Major facilitator superfamily domain, general substrate transporter	51

## Supplementary Materials

The following are available online at https://www.mdpi.com/2076-2607/7/10/390/s1, Figure S1: Hydropathy profile of the MfsT protein amino acid sequence created by TMHMM, Figure S2: Three dimensional modeling of MfsT protein formed by SwissProt.
