# Supplementary material for: Genetic Modification of mfsT Gene Stimulating the Putative Penicillin Production in Monascus ruber M7 and Exhibiting the Sensitivity towards Precursor Amino Acids of Penicillin Pathway
Source: Microorganisms. 2019 Sep 24;7(10):390. doi: 10.3390/microorganisms7100390 (PMC6843564; doi:10.3390/microorganisms7100390)
Supplement: Supplementary file 1 [file microorganisms-07-00390-s001.zip › microorganisms-579594-supplementary.pdf]

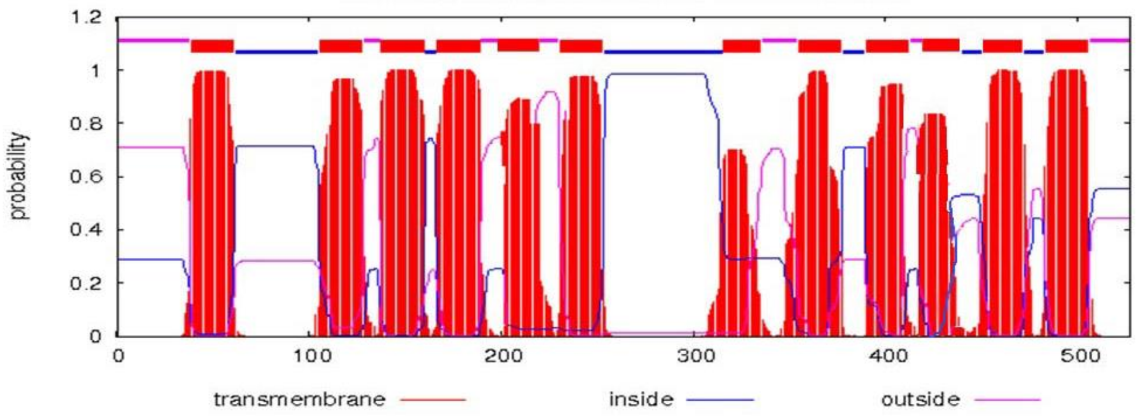

Figure S1. Hydropathy profile of the MfsT protein amino acid sequence created by TMHMM. The TMSs are predicted by using (http://www.cbs.dtu.dk/services/TMHMM/), displaying the 12 clear hydrophobic transmembrane spanning domains in Mfst protein.

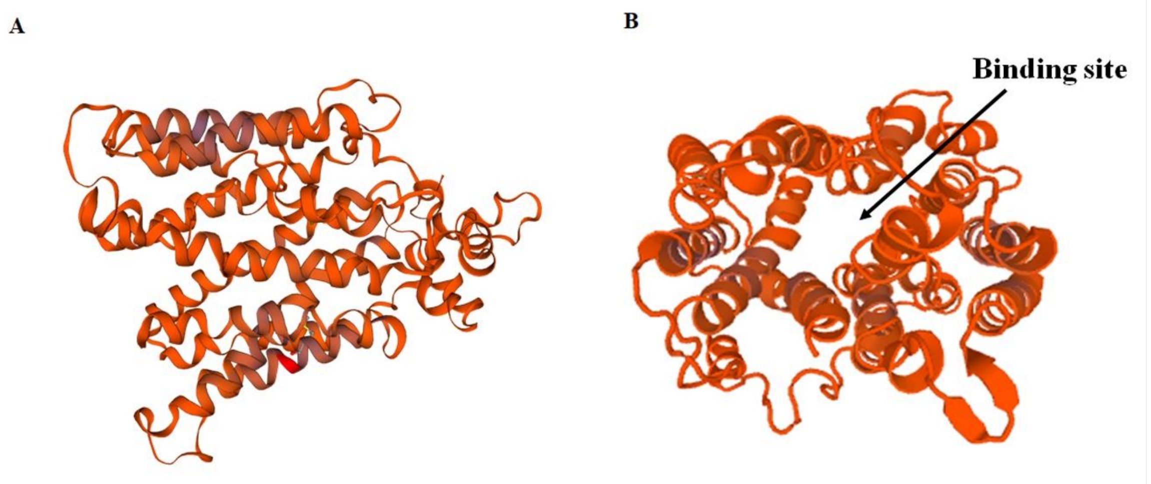

Figure S2. Three dimensional modeling of MfsT protein formed by SwissProt. (**A**) Frontal view; (**B**) Sagital view
